# Supplementary material for: Germline Cas9 promoters with improved performance for homing gene drive
Source: Nat Commun. 2024 May 29;15:4560. doi: 10.1038/s41467-024-48874-1 (PMC11137117; doi:10.1038/s41467-024-48874-1)
Supplement: Supplementary file 1 — Supplementary Information [file 41467_2024_48874_MOESM1_ESM.pdf]

Supplemental Information

Germline Cas9 promoters with improved performance for homing gene drive

Jie Du[1*], Weizhe Chen[1,2], Xihua Jia[1], Xuejiao Xu[1], Emily Yang[3], Ruizhi Zhou[1], Yuqi Zhang[1], Matt Metzloff[3], Philipp W. Messer[3], Jackson Champer[1*]

[1]Center for Bioinformatics, School of Life Sciences, Center for Life Sciences, Peking University, Beijing, China 100871
[2]School of Life Sciences, Tsinghua University, Beijing, China 100084
[3]Department of Computational Biology, Cornell University, Ithaca, NY 14853

*JD (dujie123@pku.edu.cn), JC (jchamper@pku.edu.cn)

A Split drive "reverse" Cas9 elements

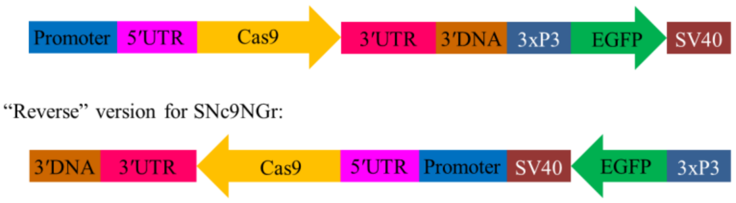

B EGFP target drive

Phenotype	Genotypes
R	D/D, D/r2,
RG	D/+, D/r1
WT	r2/r2
G	+/+, +/r1, +/r2, r1/r1, r1/r2, +/rm, r1/rm
Rm	D/rm
m	r2/rm, rm/rm

C Split drive

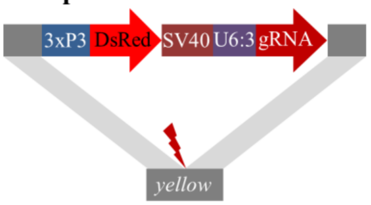

Phenotype	♂ genotypes	♀ genotypes
Ry	D	D/D, D/r2,
R	none	D/+, D/r1
y	r2	r2/r2
WT	+, r1	+/+, +/r1, +/r2, r1/r1, r1/r2, +/rm, r1/rm
Rm	none	D/rm
m	rm	r2/rm, rm/rm

D Split modification rescue drive

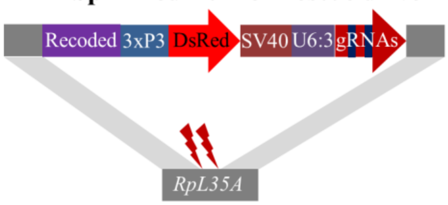

Phenotype	Genotypes
R	D/D, D/+*
WT	+/+
Nonviable	D/r2, D/rm, +/r2, +/rm, r2/r2, r2/rm, rm/rm

*Could be nonviable or unhealthy depending on somatic Cas9 expression

E Split suppression drive

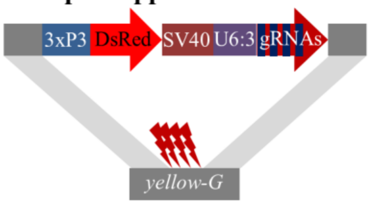

Phenotype	Genotypes
R, ♀ sterile	D/D, D/r2
R, ♀ low fertility	D/rm
R	D/+*
WT, ♀ sterile	r2/r2
WT, ♀ low fertility	r2/rm, rm/rm?
WT	+/+, +/r2, +/rm

*Could be low fertility or sterile depending on Cas9 expression

R - DsRed, G - EGFP, y - yellow, WT - wild-type, m - yellow mosaic
D - drive allele, r1 - functional resistance allele (only observed for 1-gRNA drive),
r2 - nonfunctional resistance allele, rm - mosaic for resistance alleles, + - wild-type allele (or original EGFP)

Figure S1 Schematic diagram of additional constructs with rearranged elements. (A) In some split Cas9 lines, the orientation of the Cas9 gene is reversed (on in one case, the orientation of the EGFP gene) to prevent interference between the promoter elements of Cas9 and EGFP. (**B**) Drives targeting EGFP are marked with DsRed. The drive and nonfunctional resistance alleles (r2) can disrupt EGFP. Resistance alleles can also be mosaic. Functional resistance alleles (r1) are rare. (**C**) The *yellow* gene has a recessive knockout phenotype that produces a yellow color on the body and wings. Both the drive and nonfunctional resistance alleles can produce this knockout phenotype. The drive also carries a DsRed gene and is designed to be used with a split Cas9 line. Because *yellow* is on the X-chromosome, males have simpler genotypes, while female phenotypes are more complex and influenced by factors such as leaky somatic Cas9 expression and maternal Cas9 deposition. This drive produces perhaps ~10% functional resistance alleles. (**D**) The split rescue drive targets the haplolethal gene *RpL35A*. Flies with nonfunctional resistance alleles are nonviable. Somatic Cas9 expression can result in resistance allele formation or drive conversion, so higher amounts are needed to reduce viability. (**E**) The split suppression drive targets and disrupts *yellow-G*, a haplosufficient female fertility gene. Thus, only females with wild-type alleles are fertile. Somatic Cas9 expression substantially reduces fertility.

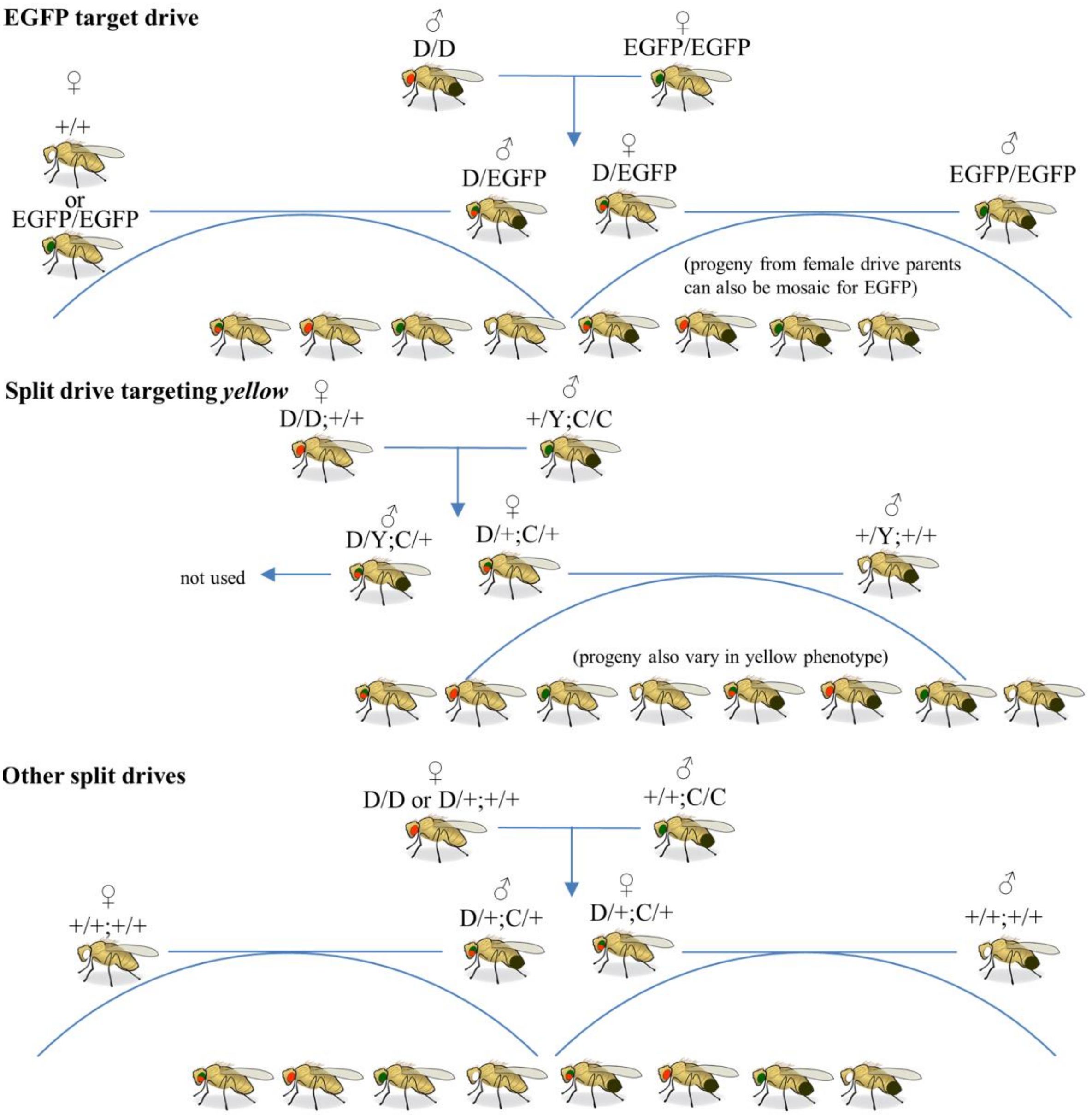

Figure S2 Crossing scheme. The figure shows the crossing scheme for each class of drive in the study. Because maternal Cas9 and gRNA generate embryo resistance alleles, only drive males were used to start the crosses for the EGFP target drive so that drive heterozygous offspring would all be drive/wild-type (instead of potentially drive/resistance). The split drives were not affected by this issue because gRNA is also required to be maternally deposited for embryo resistance alleles to form. For the split drive targeting *yellow*, only female heterozygotes were assessed for drive performance because the drive is X-linked.

Table S1 List of donor plasmid names with Cas9 regulatory features

SN = split Cas9 line, BHD = drive targeting EGFP, *except for BHDaaN, which is split Cas9 at site A

Line/Donor Plasmid	Promoter/5′ UTR	3′ UTR/Terminator	Other Feature
SNc9VnG	*vasa*	*nanos*	
SNc9VnGp	*vasa*	*nanos*	PEST
SNc9NG	*nanos*	*nanos*	
SNc9NGr	*nanos*	*nanos*	reverse
SNc9NsG	*nanos*	*shu*	
SNc9NvG	*nanos*	*vasa*	
BHDgN1cv3[10]	*nanos*	*nanos*	
BHDgN1p	*nanos*	*nanos*	PEST
BHDaaN*[20]	*nanos*	*nanos*	*split Cas9 line
SNc9DnG	*rcd-1r*	*nanos*	
SNc9DG	*rcd-1r*	*rcd-1r*	
SNc9DsG	*rcd-1r*	*shu*	
SNc9DpG	*rcd-1r*	*nanos*	PEST
SNc9DpGr	*rcd-1r*	*nanos*	reverse, PEST
BHDgD1	*rcd-1r*	*nanos*	
BHDgD1d	*rcd-1r*	*rcd-1r*	
SNc9XnGr	*CG4415*	*nanos*	reverse
SNc9XG	*CG4415*	*CG4415*	
SNc9XSGr1	*CG4415*	*shu*	reverse, line 1
SNc9XSGr2	*CG4415*	*shu*	reverse, line 2
SNc9XGd	*CG4415*	*rcd-1r*	
SNc9XpG	*CG4415*	*nanos*	reverse, PEST
SNc9XpGv2	*CG4415*	*nanos*	PEST
SNcc9XpG	*CG4415*	*nanos*	PEST, site C
SNcc9XnG	*CG4415*	*nanos*	site C
BHDgX1	*CG4415*	*nanos*	
BHDgX1x	*CG4415*	*CG4415*	
SNc9ZG	*zpg*	*zpg*	
BHDgZ1	*zpg*	*nanos*	
BHDgZ1z	*zpg*	*zpg*	
SNc9SnG	*shu*	*nanos*	
SNc9SpG	*shu*	*nanos*	reverse, PEST
SNc9SpGv2	*shu*	*nanos*	PEST
BHDgS1	*shu*	*nanos*	
BHDgS1s	*shu*	*shu*	
SNc9FnG	*CG17658*	*nanos*	
SNc9FpG	*CG17658*	*nanos*	PEST
SNc9CnG	*CG7878*	*nanos*	
SNc9CpG	*CG7878*	*nanos*	PEST
SNc9EnG	*CG3223*	*nanos*	
BHDgB1	*β2-tubulin*	*nanos*	
BHDgM1	*mei-W68*	*nanos*	

Table S2 Sizes of Cas9 regulatory elements

**Gene**	**Promoter**	**5′ UTR**	**3′ UTR**	**3′DNA**	**Note**
vasa	2090	151	148	427	promoter includes 1192 bp of 5′ end of *TfIIS* gene, 56599 bp intron between two 5′ UTR exons deleted
nanos	672	261	839	78	promoter includes 450 bp of 5′ end of *CG11779* gene
rcd-1r	1946	146	165	41	
CG4415	827	133	191+72 intron	56	
zpg	262	123	308	125	promoter includes 116 bp of *Rexo5* 5′ UTR
shu	436	92	234	88	promoter includes 240 bp of *Snap29* 3′ UTR
CG17658	212	140+58 intron	no	N/A	promoter includes 137 bp of possible *upf3* 5′ UTR
CG7878	726	128	N/A	N/A	promoter includes 292 bp of *puc* 3′ UTR
CG3223	349	87	N/A	N/A	promoter includes 21 bp of *CG11052* 5′ UTR
β2-tubulin	1183	230	N/A	N/A	promoter includes 1137 bp of 3′ end of *task7* gene
mei-W68	614	886+996 intron	N/A	N/A	

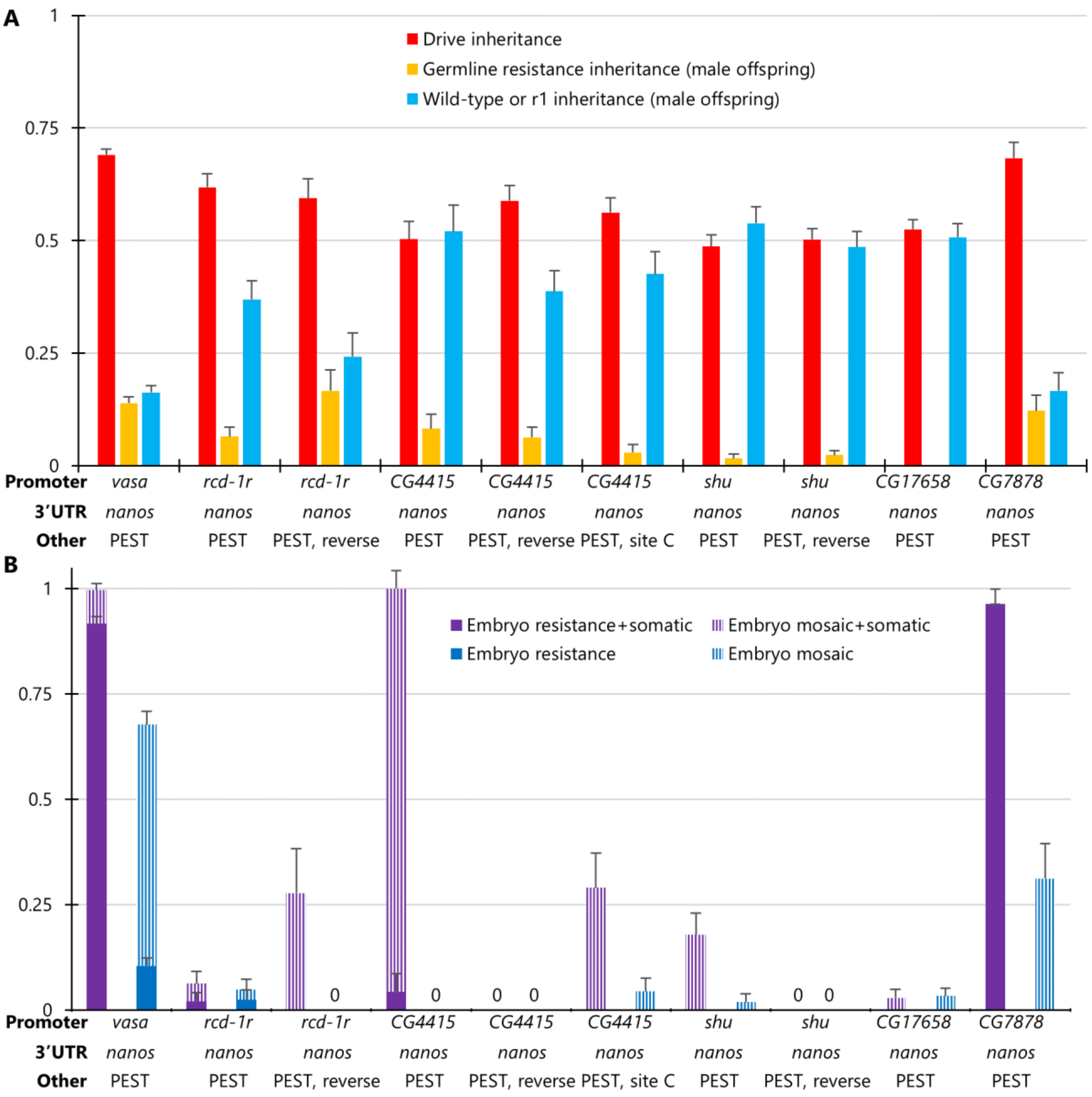

Figure S3 *yellow* target site drive performance with PEST domain. Females heterozygous for different Cas9 alleles and for the drive targeting *yellow* were crossed with w^{1118} males. (**A**) Their progeny were phenotyped for DsRed (drive), EGFP (Cas9), and yellow body color. The germline resistance inheritance shows the fraction of male progeny with yellow body but no drive, and wild-type or r1/functional resistance indicates the fraction of wild-type male offspring. (**B**) The fraction of offspring with yellow phenotype inheriting the drive and also Cas9 is "Embryo resistance+somatic" because maternally deposited Cas9 /gRNA and somatic expression could cause the yellow phenotype. "Embryo resistance rate" reports the fraction of drive offspring lacking Cas9 that have the yellow phenotype, which must be from maternal deposition. "Reverse" indicates a change in orientation on one gene so that the Cas9 promoter and 3xP3 of EGFP are not adjacent. Error bars represent SEM. Source data provided in Data Set S3.

Table S3 Maximum likelihood estimates of female drive fitness

AICc - Akaike information criterion, corrected
Brackets show 95% confidence intervals

Cage 1 *rcd-1r* promoter

Model	Log-Likelihood	AICc	Effective Population	Female D/+ Fitness
Normal	15.5	-24.5	8.3% [2.4%, 19.2%]	0.29 [0.09, 0.54]

Cage 2 *CG4415* promoter with reverse orientation

Model	Log-Likelihood	AICc	Effective Population	Female D/+ Fitness
Normal	15.2	-25.2	4.3% [1.5%, 8.7%]	0.59 [0.28, 1.02]
Generations 4-5 removed*	12.3	-19.5	5.2% [1.8%, 11.3%]	0.84 [0.43, 1.44]

*Removed because food was likely drier in these generational transitions than for others

Cage 3 *CG4415* promoter with reverse orientation

Model	Log-Likelihood	AICc	Effective Population	Female D/+ Fitness
Normal	17.9	-30.18	15.0% [5.0%, 32.1%]	0.28 [0.11, 0.49]

Cage 4 *CG4415* promoter with reverse orientation

Model	Log-Likelihood	AICc	Effective Population	Female D/+ Fitness
Normal	14.1	-22.7	5.0% [1.5%, 10.5%]	0.56 [0.30, 0.91]
Last generation removed*	16.2	-26.7	8.0% [2.8%, 17.5%]	0.58 [0.37, 0.86]

*Removed because of very low population size and large carrier frequency fluctuation

Combined cages normal food *CG4415* promoter with reverse orientation

Model	Log-Likelihood	AICc	Effective Population	Female D/+ Fitness
Cage 2 - gen4,5 & Cage 4 - last gen	28.1	-51.4	6.9% [3.3%, 12.5%]	0.66 [0.46, 0.91]

Combined cages dry food *CG4415* promoter with reverse orientation

Model	Log-Likelihood	AICc	Effective Population	Female D/+ Fitness
Cage 2 gen3-6 & Cage 3	32.4	-60.2	5.8% [2.8%, 9.7%]	0.36 [0.21, 0.54]

Cage 5 *CG4415* promoter at Site C

Model	Log-Likelihood	AICc	Effective Population	Female D/+ Fitness
Normal	4.79	6.41	4.6% [~0, 12.8%]	>3* [1.569, >3]

*The drive flies had an advantage in this case due to unknown causes, not likely related to actual drive-associated factors - this cage is excluded from the combined analysis

Cage 6 *CG4415* promoter at Site C

Model	Log-Likelihood	AICc	Effective Population	Female D/+ Fitness
Normal	1.6	-11.27	10.3% [* , 51.0%]	1.146 [*, *]

*Could not be obtained due to low numbers of generational transitions

Cage 7 *CG4415* promoter at Site C

Model	Log-Likelihood	AICc	Effective Population	Female D/+ Fitness
Normal	7.4	-8.45	6.4% [1.7%, 15.0%]	1.43 [0.56, 3.34]
Last generation removed*	11.0	-15.0	19.2% [5.1%, 46.7%]	1.51 [0.91, 2.44]

*Removed because of very low population size and large carrier frequency fluctuation

Combined cages *CG4415* promoter at Site C

Model	Log-Likelihood	AICc	Effective Population	Female D/+ Fitness
Cage 6 & Cage 7 - last gen	12.48	-19.0	16.5% [5.0%, 37.1%]	1.48 [0.87, 2.47]
